# Supplementary material for: Quantifying yield losses from Bt resilience among maize cultivars in South Africa
Source: Nat Commun. 2026 Apr 1;17:4704. doi: 10.1038/s41467-026-71156-x (PMC13212967; doi:10.1038/s41467-026-71156-x)
Supplement: Supplementary file 1 — Supplementary Information [file 41467_2026_71156_MOESM1_ESM.pdf]

Supplementary Figures and Tables for Quantifying Yield Losses from Bt Resilience among Maize Cultivars in South Africa

Figure SA1. Estimated yield gains for stacked trait cultivars relative to conventional for three subsamples of the data. 2005 is when stacked cultivars first appear in-trial, 2008 is when Bt resistance is first reported, and 2013 is when new cultivars were released to combat this resistance. Bars represent a 95% confidence interval that is robust to spatial correlation.

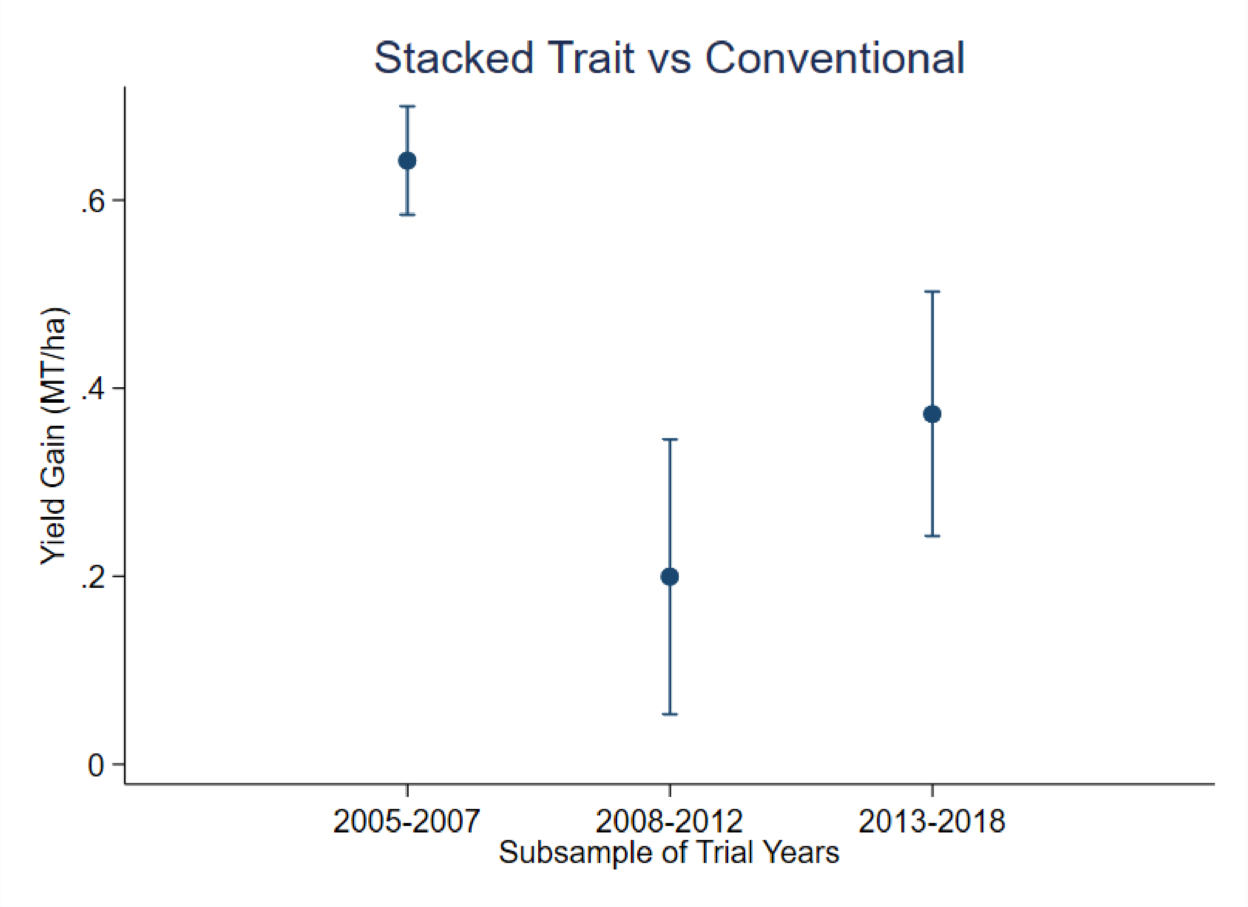

Figure SA2. Estimated GM effect as a function of the cultivars commercial release year. (a) compares single-trait HT cultivars to conventional; (b) compares stacked cultivars to single-trait Bt. Bars represent a 95% confidence interval that is robust to spatial correlation.

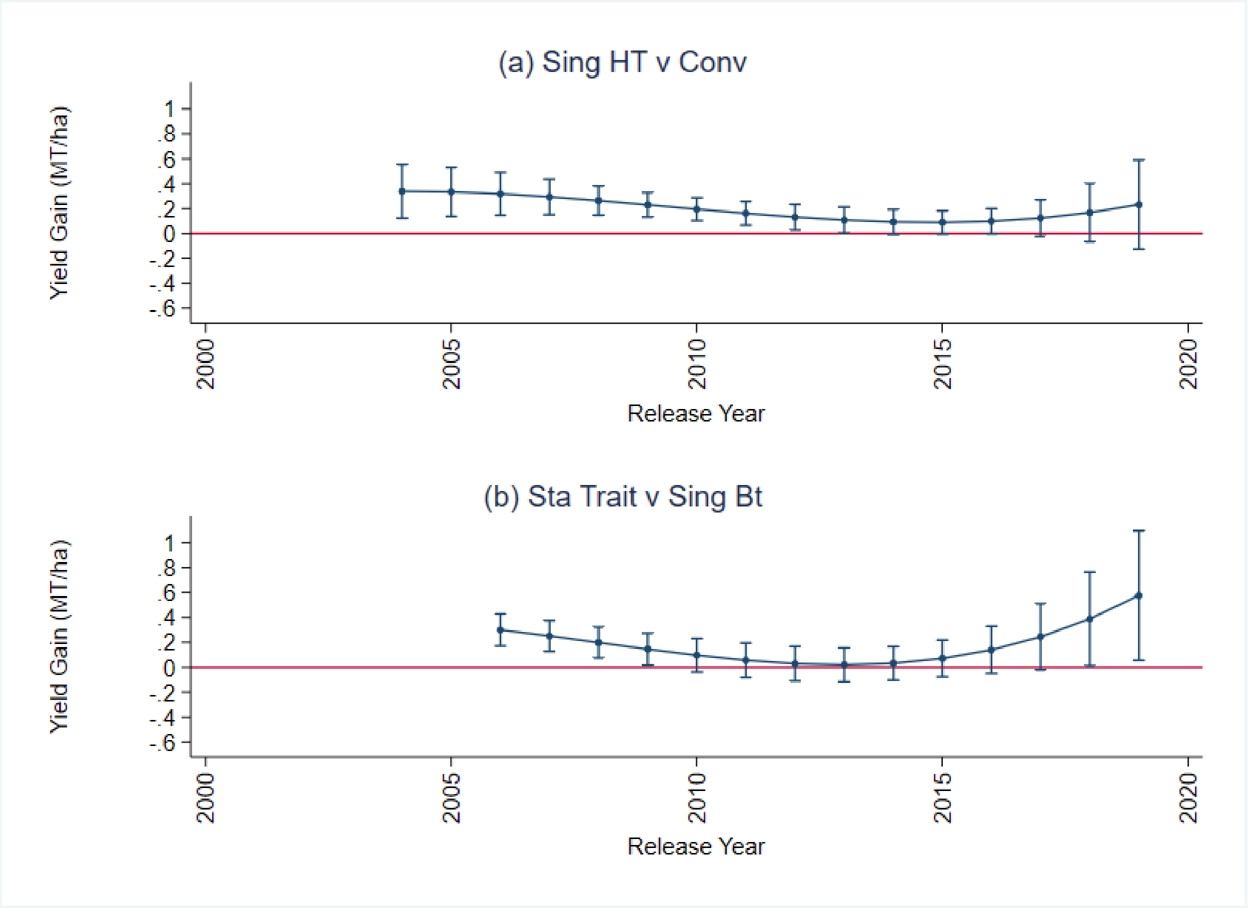

Figure SA3. Evolution of GM effect in the Free State Province. This replicates Figure 4 using only trials in Free State. Bars represent a 95% confidence interval that is robust to spatial correlation.

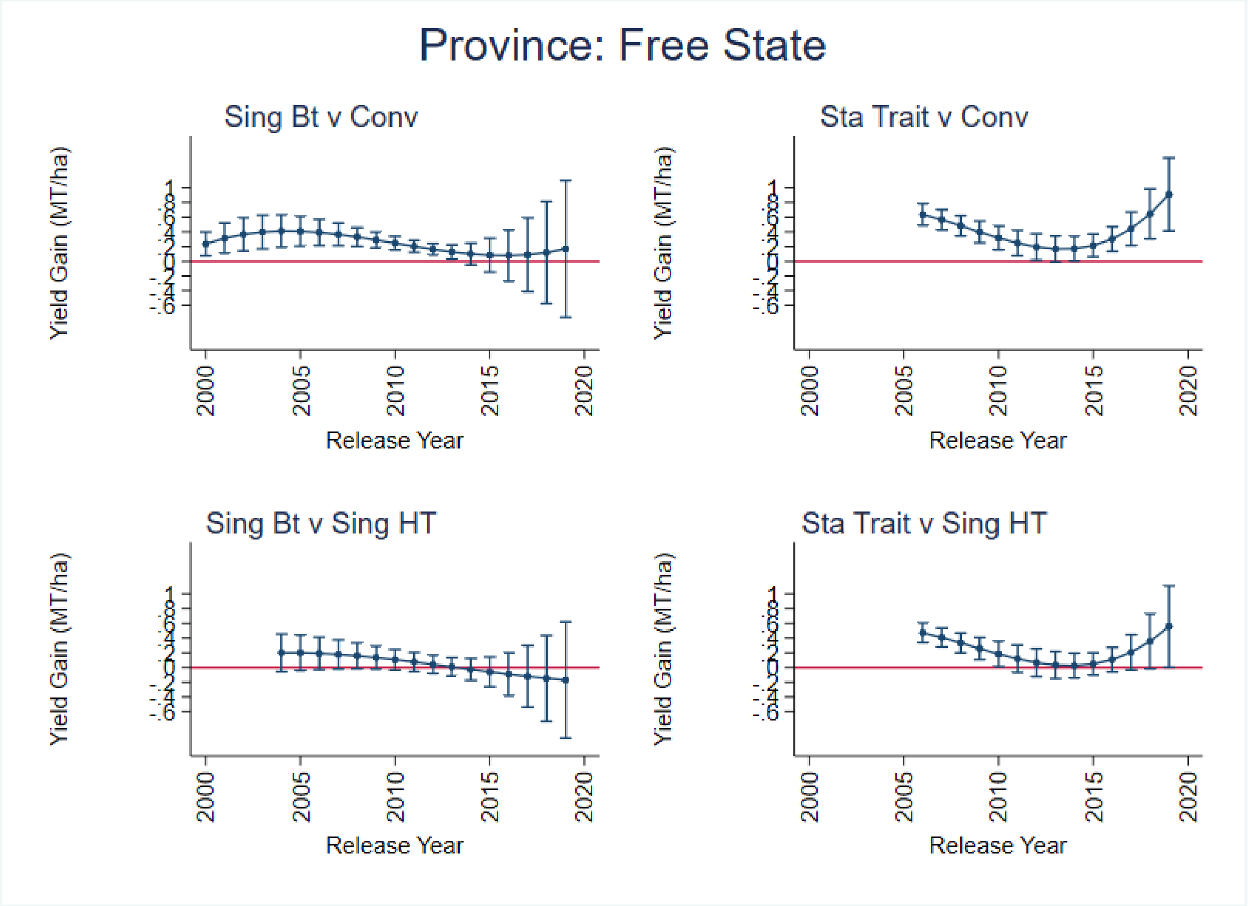

Figure SA4. Evolution of GM effect in the Gauteng Province. This replicates Figure 4 using only trials in Gauteng. Bars represent a 95% confidence interval that is robust to spatial correlation.

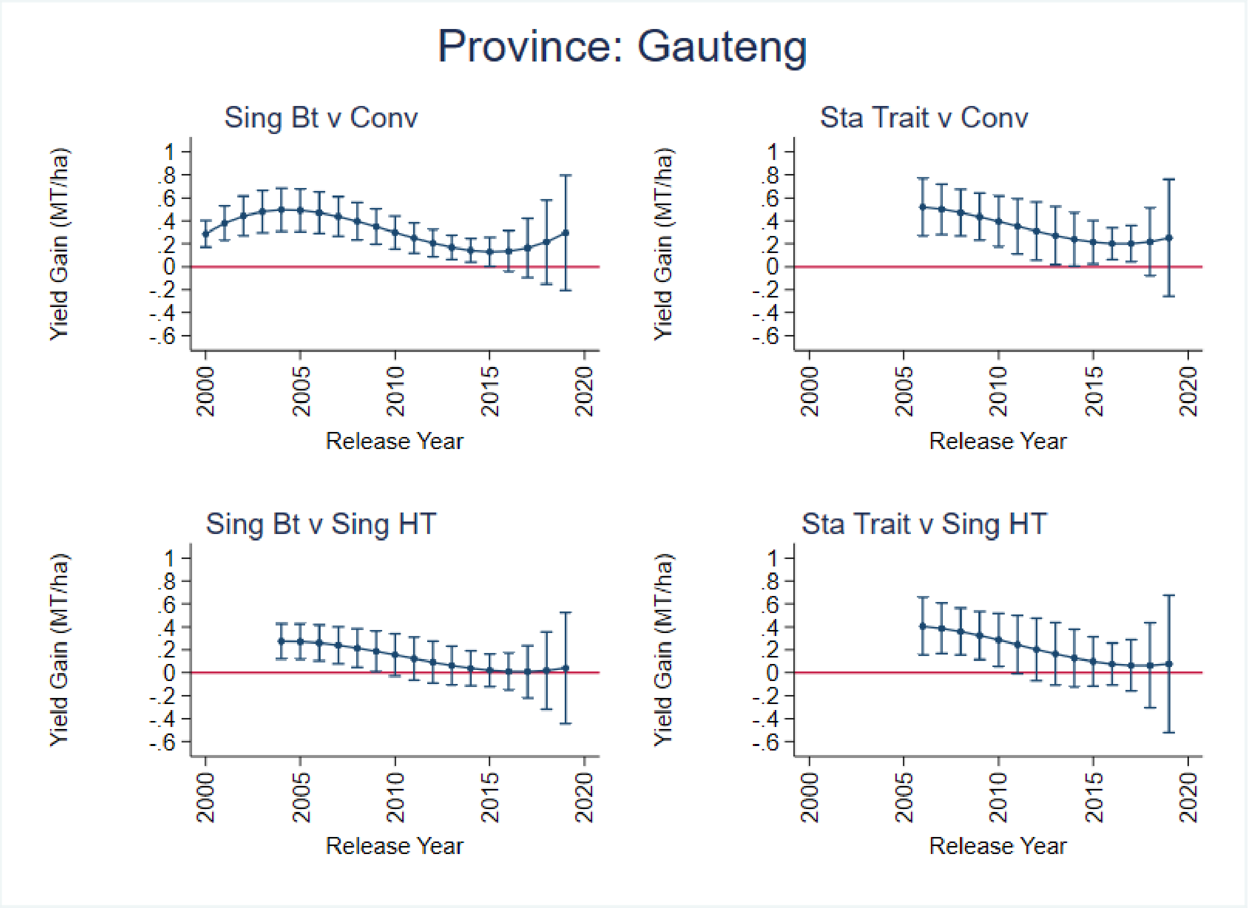

Figure SA5. Evolution of GM effect in the KwaZulu-Natal Province. This replicates Figure 4 using only trials in KwaZulu-Natal. Bars represent a 95% confidence interval that is robust to spatial correlation.

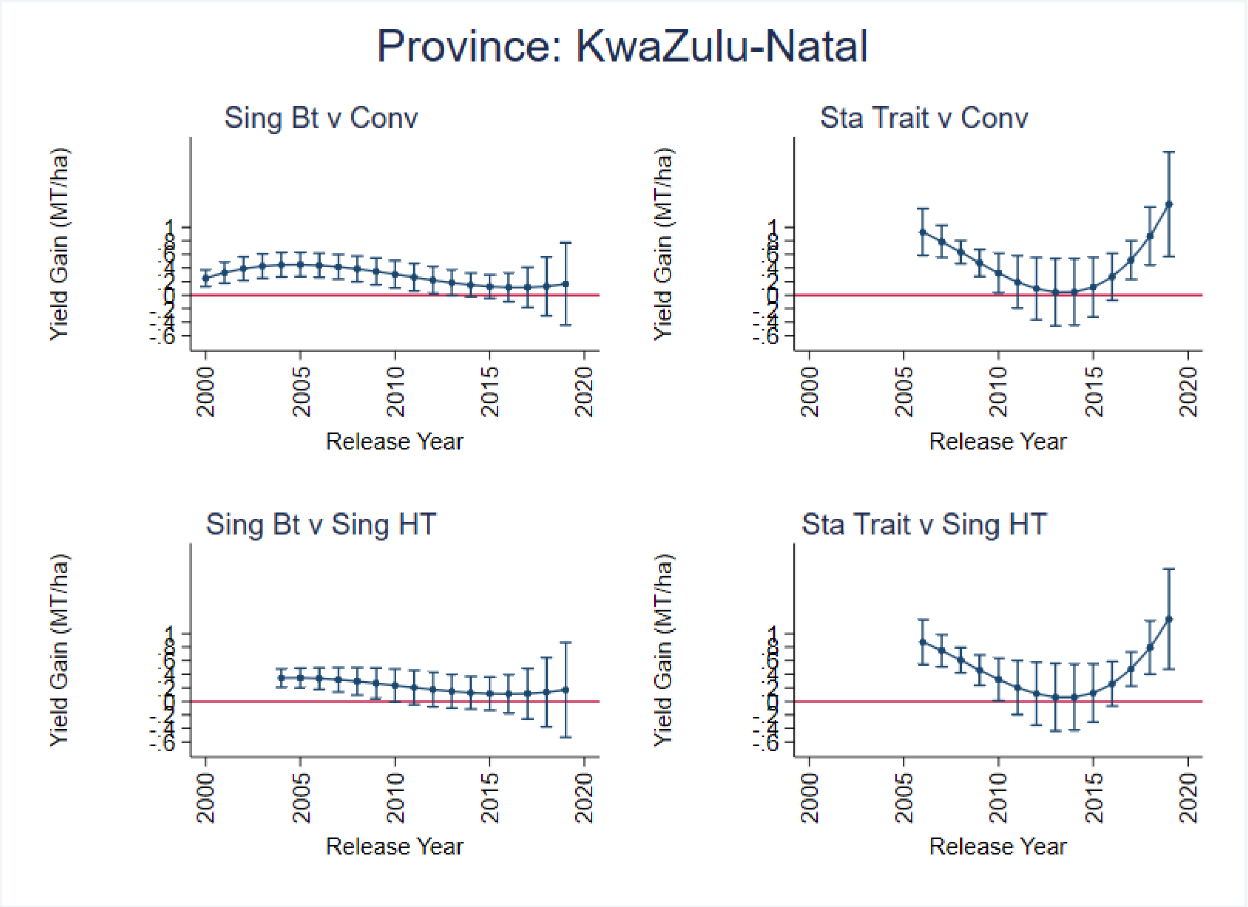

Figure SA6. Evolution of GM effect in the Mpumalanga Province. This replicates Figure 4 using only trials in Mpumalanga. Bars represent a 95% confidence interval that is robust to spatial correlation.

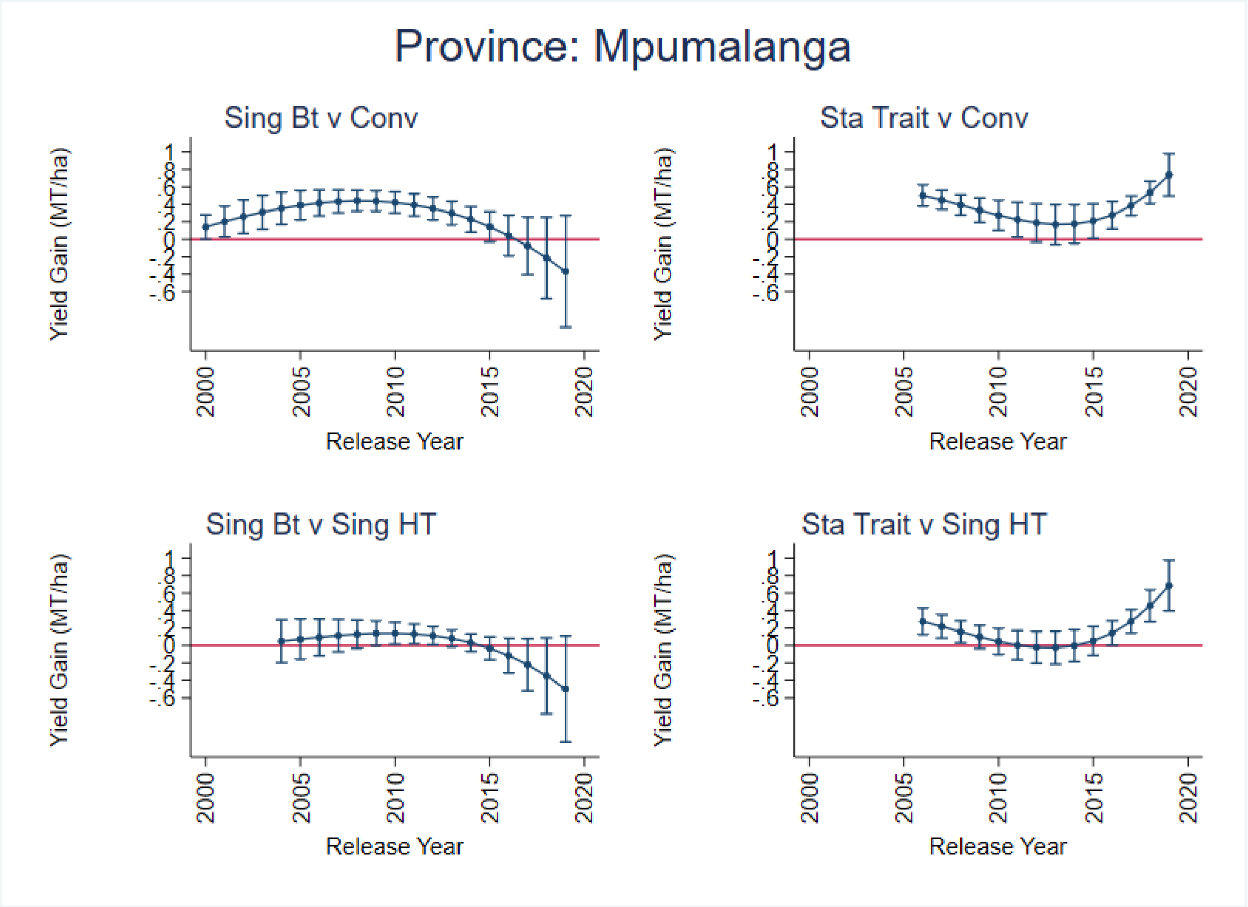

Figure SA7. Evolution of GM effect in the North West Province. This replicates Figure 4 using only trials in North West. Bars represent a 95% confidence interval that is robust to spatial correlation.

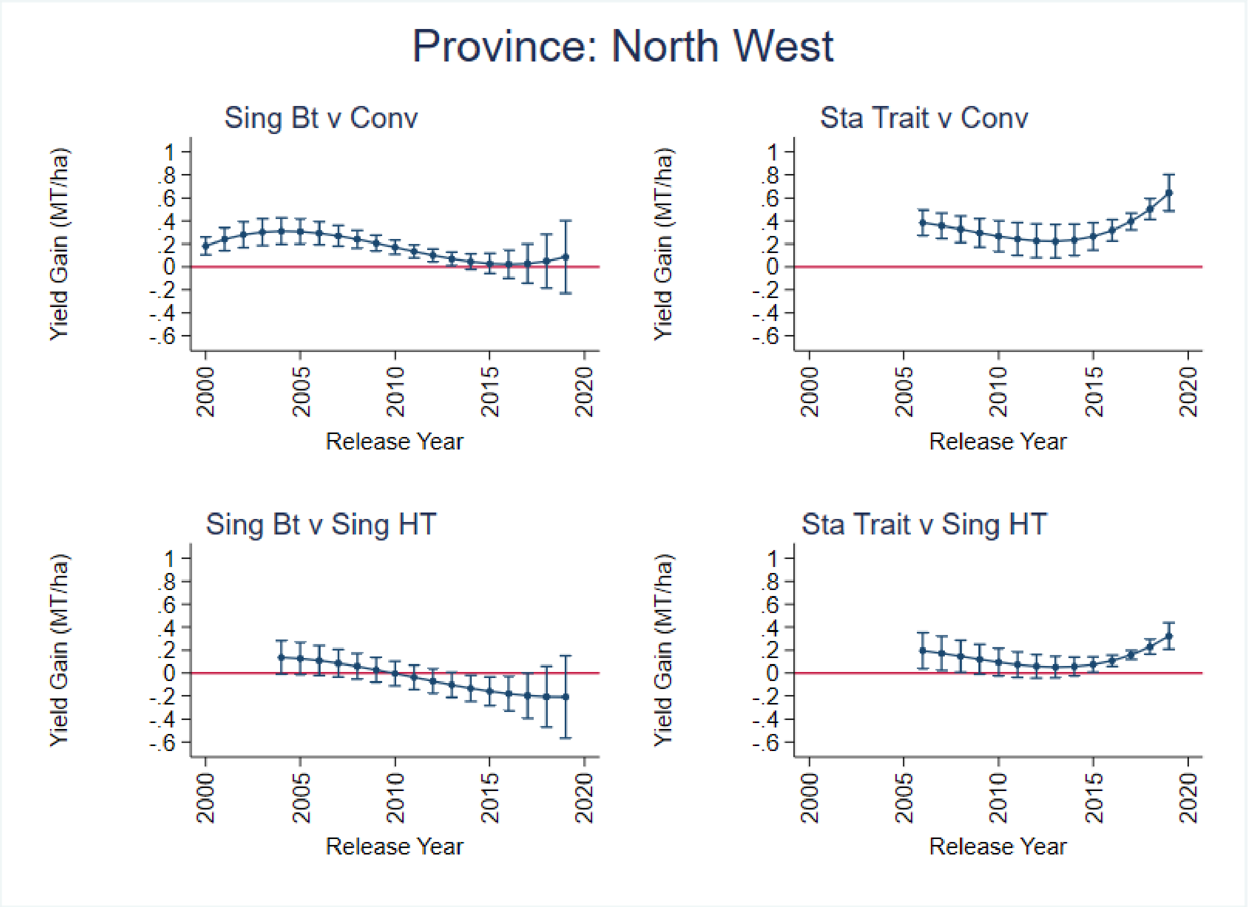

Figure SA8. Evolution of GM effect using a multilevel mixed effects model. The GM effect is allowed to vary with location, release year, and trial year. The left column averages the effect across trial year and reports the effect by location and release year; while the right column averages across release year and reports the effect by location and trial year.

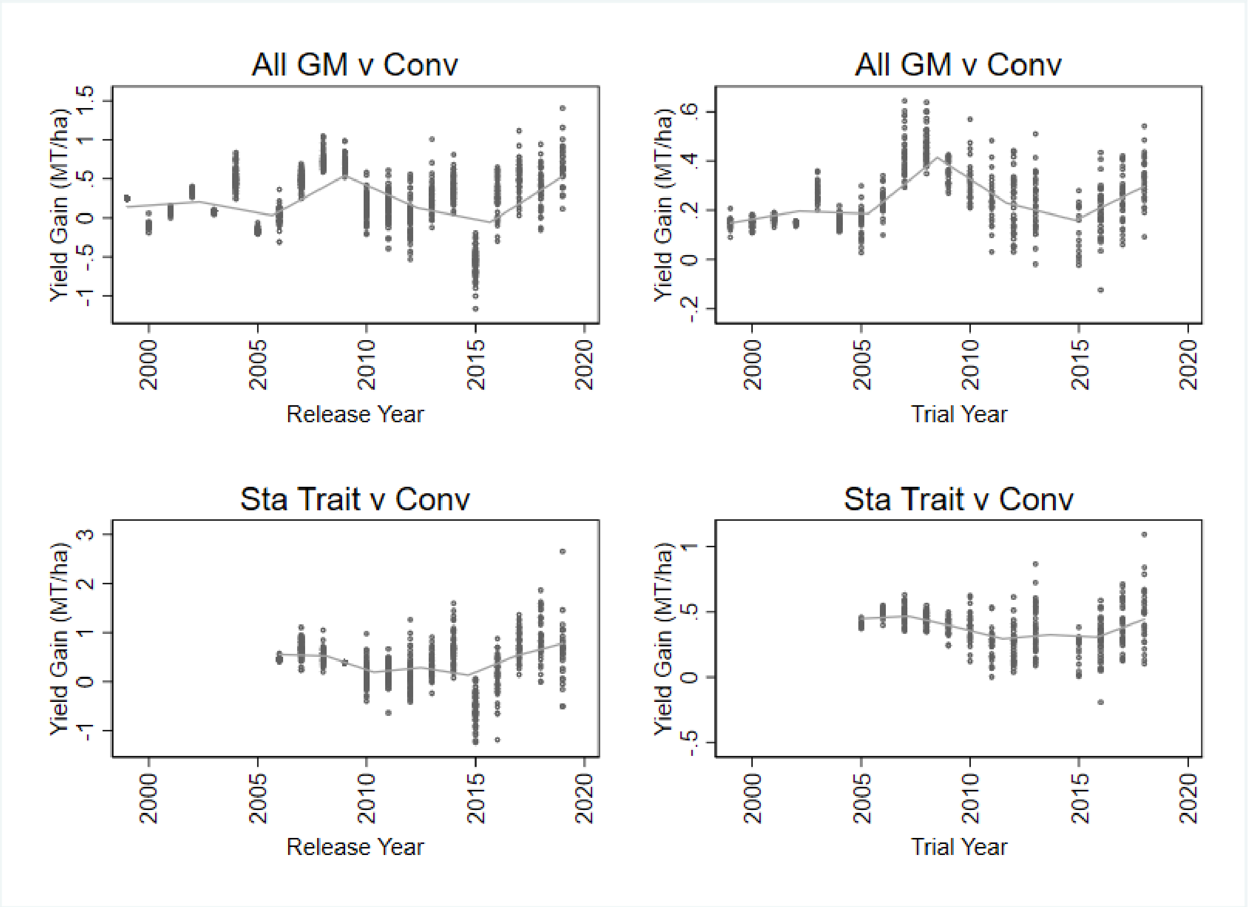

Figure SA9. Estimated GM effect as a function of the field trial year. Bars represent a 95% confidence interval that is robust to spatial correlation.

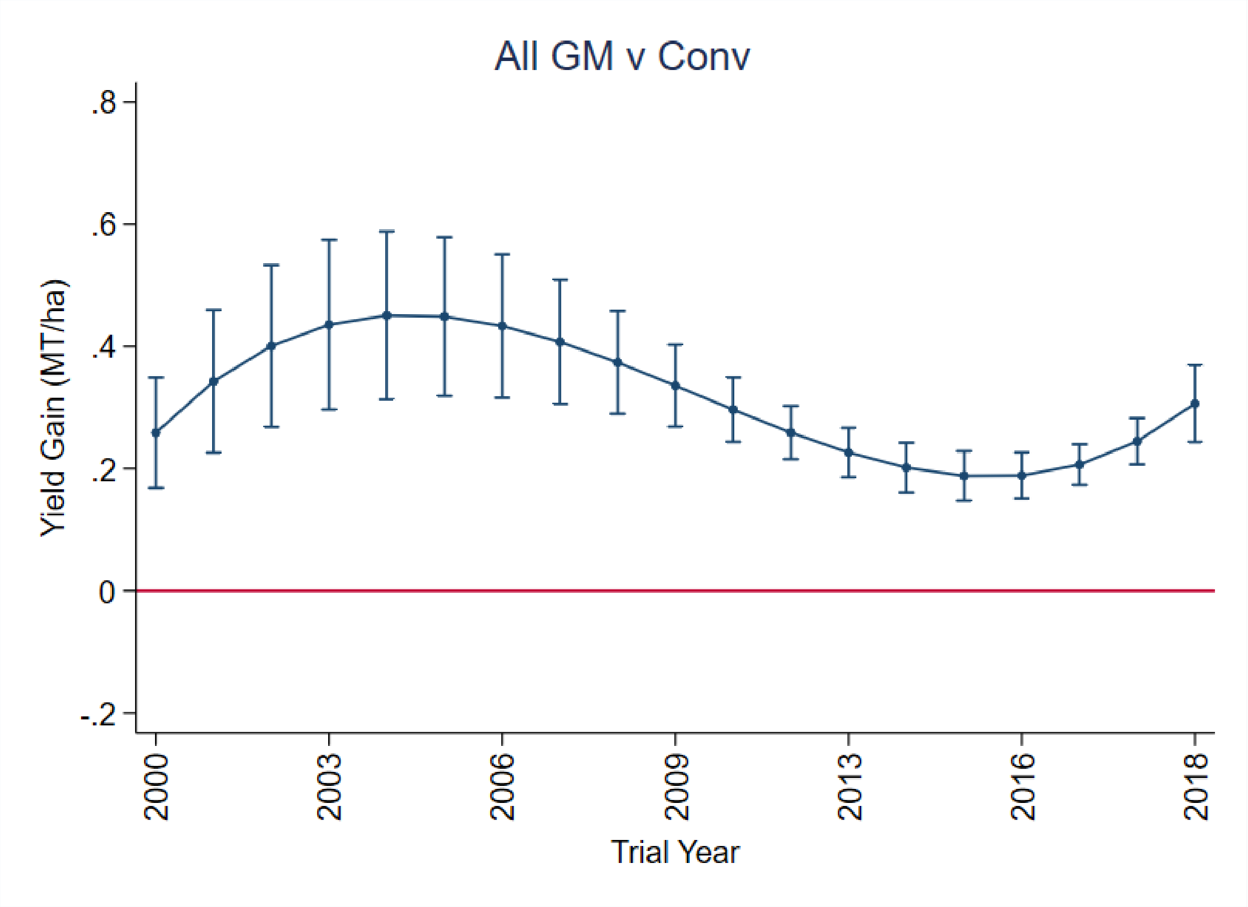

Figure SA10. Number of trials for conventional, single Bt, single HT, and stacked (both trait) cultivars.

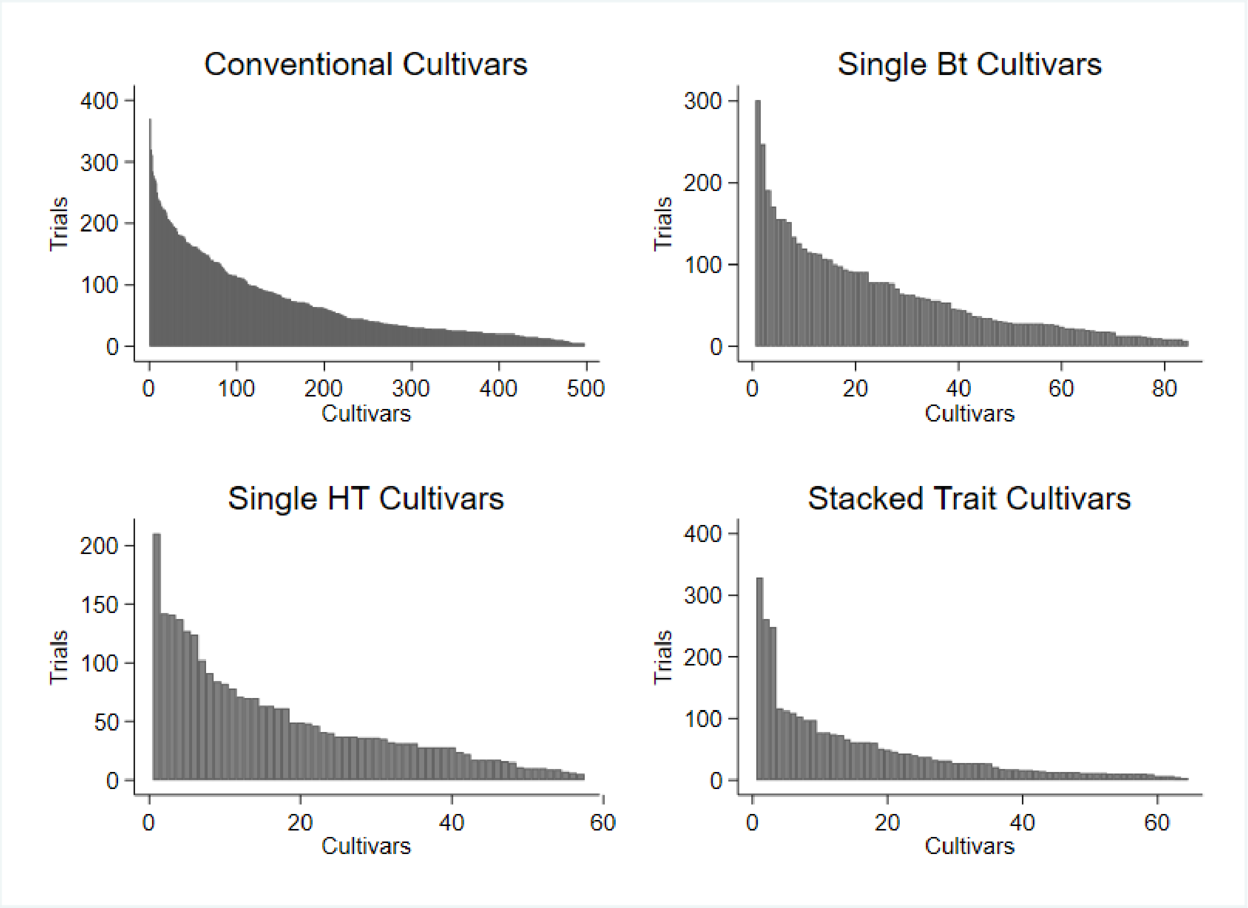

Figure SA11. Release years represented by trial year for (a) conventional, (b) single Bt, (c) single HT, and (d) stacked (both Bt and HT) cultivars.

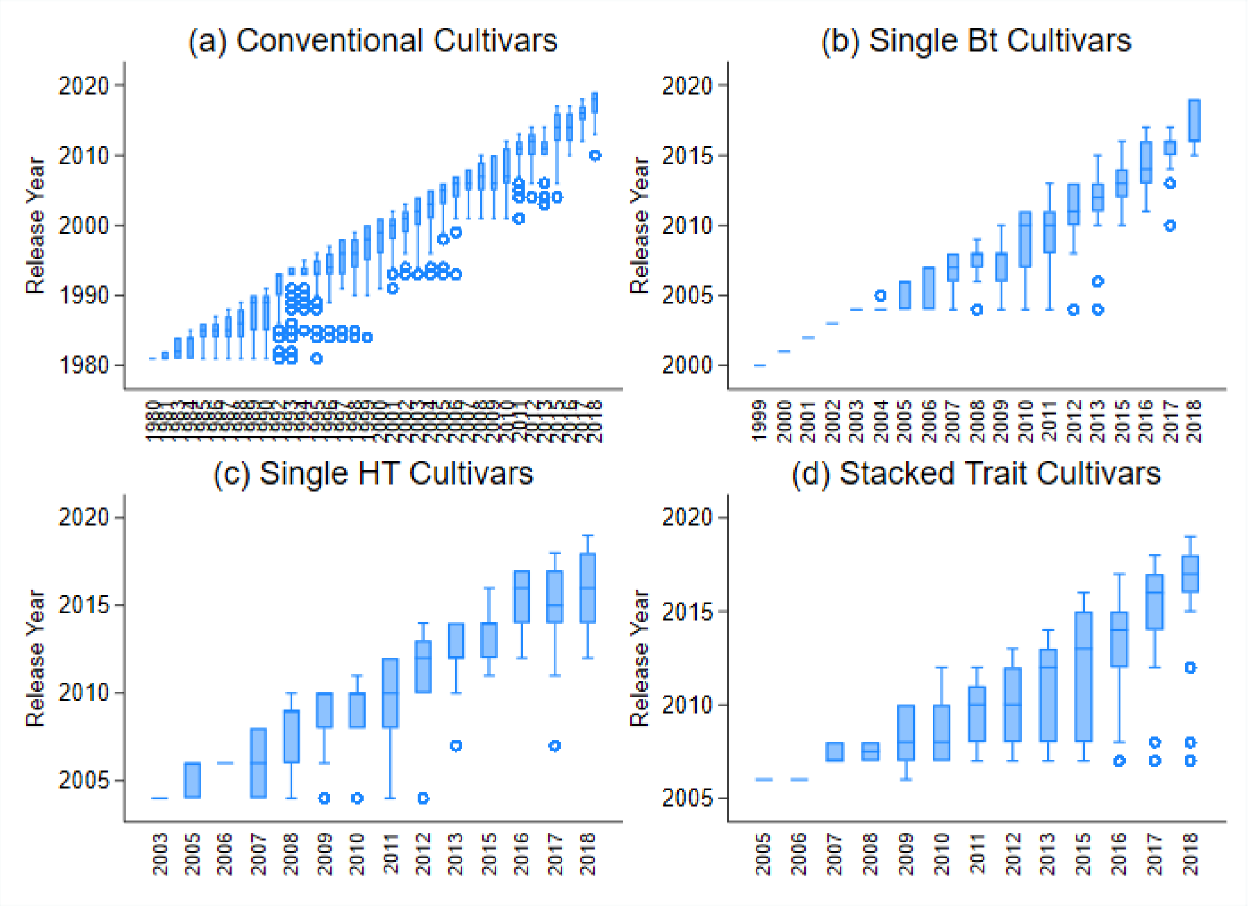

Table SA1. Estimates of GM yield gains evolving over time

Year Ending	Estimate	Std Error	R^2	Nobs	N Years	N Loc
2005	0.202*	0.092	0.83	35,351	24	79
2006	0.233***	0.061	0.83	36,842	25	84
2007	0.368***	0.096	0.84	38,830	26	87
2008	0.394***	0.064	0.84	41,124	27	90
2009	0.363***	0.064	0.84	42,974	28	90
2010	0.366***	0.039	0.83	49,238	29	91
2011	0.333***	0.042	0.81	54,600	30	93
2012	0.308***	0.041	0.82	60,265	31	94
2013	0.291***	0.038	0.81	66,938	32	94
2015	0.277***	0.037	0.79	69,821	33	102
2016	0.266***	0.034	0.78	75,340	34	103
2017	0.263***	0.031	0.77	80,499	35	104
2018	0.271***	0.029	0.77	85,133	36	104

Notes: All models include location-by-year fixed effects. The year column indicates the subsample of the data used to estimate the GM effect, with the reported number indicating the final year in the subsample 1980-XX. For example, the row 2010 reports the effect from a model that is estimated using data from 1980 to 2010. Standard errors clustered by year. ***, **, and * denote statistical significance at the .1, 1, and 5 percent levels for a two-sided t-test, no adjustments were made for multiple comparisons.

Table SA2. Parameter estimates of GM effect varying across time and traits

	M1	M2	M3
Common GM Effect	0.2712***		
GM X release year		0.1700***	
GM X (release year)^2		-0.0190***	
GM X (release year)^3		0.0001***	
Single Bt X release year			0.0502
Single Bt X (release year)^2			-0.0057
Single Bt X (release year)^3			0.0001
Single HT X release year			0.0485
Single HT X (release year)^2			-0.0080
Single HT X (release year)^3			0.0002
Stacked X release year			0.1567***
Stacked X (release year)^2			-0.0215***
Stacked X (release year)^3			0.0007***
GM: P-value of joint release year effect		0.0000	
Single Bt: P-value of joint release year effect			0.0000
Single HT: P-value of joint release year effect			0.0284
Stacked: P-value of joint release year effect			0.0000
Cumulative: P-value of all joint release year effect			0.0000
R2	0.7672	0.7676	0.7681
Nobs	85,132	85,132	85,132

Notes: All models include location-by-year fixed effects. M1 assumes a static GM effect across traits, M2 allows the effect to vary dynamically by release year, and M3 further allows the dynamic effect to vary across specific traits. Standard errors clustered by year. ***, **, and * denote statistical significance at the .1, 1, and 5 percent levels where a two-sided t-test is used for parameter estimates and a two-sided F test is used for joint effects; no adjustments were made for multiple comparisons.

Table SA3. Pairwise comparisons of dynamic GM effect

	M4	M5	M6	M7
GM X release year	0.1256***	0.2463***	0.0563	0.1954***
GM X (release year)^2	-0.0121**	-0.0296***	-0.0051	-0.0245***
GM X (release year)^3	0.0003	0.0009***	0.0001	0.0008***
P-value of joint release year effect	0.0000	0.0000	0.0587	0.0000
Conventional cultivars included	Y	Y	N	N
Stacked cultivars included	N	Y	N	Y
Single Bt cultivars included	Y	N	Y	N
Single HT cultivars included	N	N	Y	Y
R2	0.7850	0.7841	0.7088	0.6957
Nobs	65,845	61,971	23,109	19,266

Notes: All models include location-by-year fixed effects. M4 is a parwise comparison of single Bt and conventional cultivars; M5 is stacked and conventional; M6 is single Bt and single HT; M7 is stacked and single HT. The effect captures the first relative to the seond, e.g. M4 estimates the yield gain of single Bt cultivars relative to conventional. Standard errors clustered by year. ***, **, and * denote statistical significance at the .1, 1, and 5 percent levels where a two-sided t-test is used for parameter estimates and a two-sided F test is used for joint effects; no adjustments were made for multiple comparisons.

Table SA4. Production losses for white maize from 2005-2018

Year	GM Yield Gain Estimate (MT/ha)	Area Sown to White Maize (1000 ha)	Total Production Loss Relative to 2004 (MT)	Consumption of Maize (kg/cap/year)	Total Loss Food Rations
2004	0.449	1,700.0	0	110.7	0
2005	0.433	1,033.0	-15,971.6	108.0	-147,844
2006	0.407	1,624.8	-67,476.6	101.1	-666,962
2007	0.374	1,737.0	-130,459.6	100.0	-1,303,944
2008	0.336	1,489.0	-168,399.6	96.6	-1,742,005
2009	0.296	1,719.7	-262,083.3	94.1	-2,783,678
2010	0.259	1,418.3	-269,368.7	101.1	-2,662,009
2011	0.226	1,636.2	-364,166.2	100.4	-3,626,070
2012	0.202	1,617.2	-399,831.8	99.4	-4,022,453
2013	0.188	1,551.2	-404,604.7	100.1	-4,042,005
2014	0.188	1,448.1	-377,297.6	101.3	-3,724,189
2015	0.189	1,014.8	-264,111.8	101.9	-2,590,601
2016	0.206	1,643.1	-398,320.4	102.4	-3,887,570
2017	0.245	1,268.1	-258,966.9	103.4	-2,504,515
2018	0.306	1,298.4	-185,089.9	96.3	-1,920,816

Notes: Yield gain estimates are from the model that estimates GM yield gains by trial year (Figure SA XX). Area sown and per-capita consumption are from Ala-Kokko et al. (2021). To calculate total production loss relative to 2004 for say, 2009, we first measure the reduction in yield gain between 2009 and 2004 and then multiple that by the area sown in 2009. Lost food rations is then that production loss divided by per-capita consumption

Table SA5. Characteristics of field trials by year

Year	# Trials	# Obs	% Conv	% Sing Bt	% Sing HT	% Stack
1980	5	240	100	0	0	0
1981	21	1,009	100	0	0	0
1983	20	1,121	100	0	0	0
1984	18	1,123	100	0	0	0
1985	20	1,127	100	0	0	0
1986	26	1,666	100	0	0	0
1987	26	1,519	100	0	0	0
1988	27	1,470	100	0	0	0
1989	24	1,507	100	0	0	0
1990	21	1,320	100	0	0	0
1992	29	2,239	100	0	0	0
1993	35	2,577	100	0	0	0
1994	13	1,071	100	0	0	0
1995	32	2,293	100	0	0	0
1996	31	2,008	100	0	0	0
1997	28	1,645	100	0	0	0
1998	24	1,664	100	0	0	0
1999	21	1,467	95.9	4.1	0	0
2000	28	1,798	97.9	2.1	0	0
2001	24	1,305	97.9	2.1	0	0
2002	21	1,129	99.2	0.8	0	0
2003	24	1,281	95.2	2.7	2.1	0
2004	30	1,563	89.4	10.6	0	0
2005	24	1,209	82.0	11.9	5.3	0.8
2006	30	1,491	76.5	18.5	4.3	0.7
2007	34	1,988	61.8	26.1	6.0	6.0
2008	36	2,294	49.3	28.6	14.0	8.0
2009	28	1,850	37.4	28.6	18.6	15.4
2010	28	6,264	37.1	38.7	11.1	13.1
2011	28	5,362	33.0	36.0	14.0	17.0
2012	32	5,665	26.4	34.9	19.1	19.6
2013	34	6,673	21.7	37.3	18.9	22.0
2015	15	2,883	30.6	28.9	19.0	21.5
2016	36	5,519	26.6	24.6	19.2	29.7
2017	33	5,159	31.3	10.6	24.8	33.3
2018	28	4,634	32.9	7.3	25.7	34.1
Total	934	85,133	60.5	16.9	10.3	12.3
1999-2018	534	59,534	43.5	24.1	14.8	17.6

Notes: % Conv reports the percentage of observations in that year that are conventional hybrids; % Sing Bt, % Sing HT, and % Stack correspond to single-trait Bt, HT, and stacked (both traits) cultivars. Trial data was not available for the years 1982, 1991, and 2014.

Table SA6. Cultivar characteristics of field trials by year

Year	# Cultivars	# Conv	# Sing Bt	# Sing HT	# Stack
1980	48	48	0	0	0
1981	48	48	0	0	0
1983	49	49	0	0	0
1984	49	49	0	0	0
1985	49	49	0	0	0
1986	49	49	0	0	0
1987	49	49	0	0	0
1988	49	49	0	0	0
1989	49	49	0	0	0
1990	49	49	0	0	0
1992	58	58	0	0	0
1993	55	55	0	0	0
1994	49	49	0	0	0
1995	55	55	0	0	0
1996	56	56	0	0	0
1997	58	58	0	0	0
1998	56	56	0	0	0
1999	58	56	2	0	0
2000	56	55	1	0	0
2001	58	57	1	0	0
2002	59	58	1	0	0
2003	62	58	3	1	0
2004	62	57	5	0	0
2005	64	52	8	3	1
2006	53	39	11	2	1
2007	62	39	17	3	3
2008	56	28	17	7	4
2009	59	21	17	11	10
2010	57	22	22	6	7
2011	59	21	20	8	10
2012	59	16	19	12	12
2013	86	18	30	14	24
2015	62	17	18	12	15
2016	60	18	13	13	16
2017	73	23	9	19	22
2018	67	22	5	17	23
Total	702	497	84	57	64
1999-2018	474	269	84	57	64

Notes: # Cultivars reports the number of in-trial cultivars. The next four columns report the number of these cultivars that are conventional hybrids, single-trait Bt, single-trait HT, or stacked (both traits). Trial data was not available for the years 1982, 1991, and 2014.

Table SA7. Out-of-sample comparison of alternative model specifications

Model	% Reduction
Release year: linear	-0.090
Release year: quadratic	-0.010
**Release year: cubic**	**0.076**
Release year: 3 knot cubic spline	0.006
Release year: 5 knot cubic spline	0.053
Trial year: linear	-0.029
Trial year: quadratic	0.000
Trial year: cubic	0.022
Trial year: 3 knot cubic spline	0.006
Trial year: 5 knot cubic spline	0.020

Notes: Root mean squared errors (RMSE) are reported as a percentage reduction relative to a baseline model with a homogenous (time invariant) GM effect. Each model is estimated 1000 times, randomly choosing 80 percent of the 85,133 observations. Parameter estimates are then used to predict yields for the omitted 20 percent in each subsample. The preferred model with a cubic temporal effect in release year is bolded.
