# Supplementary material for: Quantifying yield losses from Bt resilience among maize cultivars in South Africa
Source: Nat Commun. 2026 Apr 1;17:4704. doi: 10.1038/s41467-026-71156-x (PMC13212967; doi:10.1038/s41467-026-71156-x)
Supplement: Supplementary file 3 — Description of Additional Supplementary Files [file 41467_2026_71156_MOESM3_ESM.pdf]

File Name: Supplementary Code 1

Description:

A Stata do file that estimates the empirical models and generates the main results presented in the manuscript. The do file is named YieldRegressionsBt_MainResults and there is also a README file describing how to use it.
