# Supplementary material for: Quantifying yield losses from Bt resilience among maize cultivars in South Africa
Source: Nat Commun. 2026 Apr 1;17:4704. doi: 10.1038/s41467-026-71156-x (PMC13212967; doi:10.1038/s41467-026-71156-x)
Supplement: Supplementary file 5 — Reporting Summary [file 41467_2026_71156_MOESM5_ESM.pdf]

nature portfolio

Corresponding author(s): Jesse Tack

Last updated by author(s): Nov 12, 2025

Reporting Summary

Nature Portfolio wishes to improve the reproducibility of the work that we publish. This form provides structure for consistency and transparency in reporting. For further information on Nature Portfolio policies, see our Editorial Policies and the Editorial Policy Checklist.

Statistics

For all statistical analyses, confirm that the following items are present in the figure legend, table legend, main text, or Methods section.

n/a	Confirmed	
☐	☒	The exact sample size (*n*) for each experimental group/condition, given as a discrete number and unit of measurement
☐	☒	A statement on whether measurements were taken from distinct samples or whether the same sample was measured repeatedly
☐	☒	The statistical test(s) used AND whether they are one- or two-sided<br>*Only common tests should be described solely by name; describe more complex techniques in the Methods section.*
☐	☒	A description of all covariates tested
☐	☒	A description of any assumptions or corrections, such as tests of normality and adjustment for multiple comparisons
☐	☒	A full description of the statistical parameters including central tendency (e.g. means) or other basic estimates (e.g. regression coefficient) AND variation (e.g. standard deviation) or associated estimates of uncertainty (e.g. confidence intervals)
☐	☒	For null hypothesis testing, the test statistic (e.g. *F*, *t*, *r*) with confidence intervals, effect sizes, degrees of freedom and *P* value noted<br>*Give P values as exact values whenever suitable.*
☐	☒	For Bayesian analysis, information on the choice of priors and Markov chain Monte Carlo settings
☐	☒	For hierarchical and complex designs, identification of the appropriate level for tests and full reporting of outcomes
☐	☒	Estimates of effect sizes (e.g. Cohen's *d*, Pearson's *r*), indicating how they were calculated

Our web collection on statistics for biologists contains articles on many of the points above.

Software and code

Policy information about availability of computer code

Data collection	No software was used for data collection, Excel was used as database
Data analysis	Code run using Stata 15 MP

For manuscripts utilizing custom algorithms or software that are central to the research but not yet described in published literature, software must be made available to editors and reviewers. We strongly encourage code deposition in a community repository (e.g. GitHub). See the Nature Portfolio guidelines for submitting code & software for further information.

Data

Policy information about availability of data

All manuscripts must include a data availability statement. This statement should provide the following information, where applicable:

- Accession codes, unique identifiers, or web links for publicly available datasets
- A description of any restrictions on data availability
- For clinical datasets or third party data, please ensure that the statement adheres to our policy

The data is provided as a .dta Stata data file. The code is provided as a .do Stata do file. The code was run using Stata 15 MP.

Research involving human participants, their data, or biological material

Policy information about studies with human participants or human data. See also policy information about sex, gender (identity/presentation), and sexual orientation and race, ethnicity and racism.

Reporting on sex and gender	N/A
Reporting on race, ethnicity, or other socially relevant groupings	N/A
Population characteristics	N/A
Recruitment	N/A
Ethics oversight	N/A

Note that full information on the approval of the study protocol must also be provided in the manuscript.

Field-specific reporting

Please select the one below that is the best fit for your research. If you are not sure, read the appropriate sections before making your selection.

☐ Life sciences ☐ Behavioural & social sciences ☒ Ecological, evolutionary & environmental sciences

For a reference copy of the document with all sections, see nature.com/documents/nr-reporting-summary-flat.pdf

Ecological, evolutionary & environmental sciences study design

All studies must disclose on these points even when the disclosure is negative.

Study description	Our statistical model for estimating the yield gains associated with GM cultivars leverages field-trial-location-by-year fixed effects to control for omitted variable bias from unobserved factors that could be correlated with the appearance (or performance) of GM cultivars in the trial. The location component controls for all time-invariant factors at the location level (e.g., climate, soil quality), while the year component effects control for pest pressure, weather shocks, and non-GM technological improvements over time that are common across all cultivars. These include management improvements (e.g., increased fertilizer), widespread droughts or heat waves, "stock" germplasm improvements that are common to both conventional and GM cultivars, and evolutions of pest pressure either through a change in population or resistance to pest management strategies that include GM-specific traits. By crossing these fixed effects, note the use of "by" in the leading sentence above; we are also able to control for any of the above confounders at the location-year level. This is especially important in this context as the farm location at which the trials were conducted may or may not have been following refugia recommendations, which could in turn bias the estimated GM effect.
Research sample	We leverage a robust dataset of 85,133 field-trial maize observations spanning all major production regions in South Africa from 1980-2018 to estimate yield gains associated with the first wave of GM cultivars and explore the potential dynamic erosion of these gains since resistance was reported among this first wave of single gene Bt cultivars
Sampling strategy	The same 50 genotypes were used in all the trials and consisted of genotypes entered by the seed companies involved. Seed company entries are in order of priority. Where too many entries were received the final genotype choice was made through negotiations with each seed company.
Data collection	The yield outcomes were collected by each trial's administrator.
Timing and spatial scale	he final data for the five represented provinces contained 85,133 observations spanning 36 years (1980-2018 with no data available in 1982, 1991, nor 2014), 104 locations, and 702 cultivars.
Data exclusions	The data focused on dryland (rainfed) field trial plots.
Reproducibility	The data and code have been provided with the manuscript.
Randomization	A randomized complete block design (RCBD) with multiple replications was used throughout and each locality was allocated its trial randomization
Blinding	N/A

Did the study involve field work? ☐ Yes ☒ No

Reporting for specific materials, systems and methods

We require information from authors about some types of materials, experimental systems and methods used in many studies. Here, indicate whether each material, system or method listed is relevant to your study. If you are not sure if a list item applies to your research, read the appropriate section before selecting a response.

Materials & experimental systems

n/a	Involved in the study
☒	☐ Antibodies
☒	☐ Eukaryotic cell lines
☒	☐ Palaeontology and archaeology
☒	☐ Animals and other organisms
☒	☐ Clinical data
☒	☐ Dual use research of concern
☐	☒ Plants

Methods

n/a	Involved in the study
☒	☐ ChIP-seq
☒	☐ Flow cytometry
☒	☐ MRI-based neuroimaging

Dual use research of concern

Policy information about dual use research of concern

Hazards

Could the accidental, deliberate or reckless misuse of agents or technologies generated in the work, or the application of information presented in the manuscript, pose a threat to:

No	Yes
☒	☐ Public health
☒	☐ National security
☒	☐ Crops and/or livestock
☒	☐ Ecosystems
☒	☐ Any other significant area

Experiments of concern

Does the work involve any of these experiments of concern:

No	Yes
☒	☐ Demonstrate how to render a vaccine ineffective
☒	☐ Confer resistance to therapeutically useful antibiotics or antiviral agents
☒	☐ Enhance the virulence of a pathogen or render a nonpathogen virulent
☒	☐ Increase transmissibility of a pathogen
☒	☐ Alter the host range of a pathogen
☒	☐ Enable evasion of diagnostic/detection modalities
☒	☐ Enable the weaponization of a biological agent or toxin
☒	☐ Any other potentially harmful combination of experiments and agents

Plants

Seed stocks	South Africa Agricultural Research Council, Grain Crops Campus (ARC-GC) provided the data used in this study
Novel plant genotypes	South Africa Agricultural Research Council, Grain Crops Campus (ARC-GC) provided the data used in this study
Authentication	South Africa Agricultural Research Council, Grain Crops Campus (ARC-GC) provided the data used in this study
